# High-throughput sequencing to identify miRNA biomarkers in colorectal cancer patients

**DOI:** 10.3892/ol.2014.2215

**Published:** 2014-06-03

**Authors:** YAN SUN, LIN WANG, SHENG-CHAO GUO, XIAO-BING WU, XUE-HU XU

**Affiliations:** 1 Department of Gastroenterology, The Third Affiliated Hospital of Guangzhou Medical University, Guangzhou, Guangdong 510150, P.R. China; 2 Department of Oncology, Guangzhou Red Cross Hospital, Guangzhou, Guangdong 510150, P.R. China; 3 Department of General Surgery, The Third Affiliated Hospital of Guangzhou Medical University, Guangzhou, Guangdong 510150, P.R. China

**Keywords:** colorectal cancer, high-throughput sequencing, miRNA, biomarker

## Abstract

The altered expression of microRNAs (miRNAs) is associated with a number of cancer types. The study of the association between the miRNA profile and cancer may be useful to identify potential biomarkers of certain types of cancer. In the present study, 19 miRNAs were identified by high-throughput sequencing in the serum of colorectal cancer (CRC) patients. A network analysis was performed based on a computational approach to identify associations between CRC and miRNAs. The present study may be useful to identify cancer-specific signatures and potentially useful biomarkers for the diagnosis of CRC. The network analysis of miRNA-target genes may aid in identifying altered miRNA regulatory networks that are involved in tumor pathogenesis.

## Introduction

Colorectal cancer (CRC) is an important contributor to cancer-related mortality and morbidity. Accumulated data has uncovered several critical genes and pathways important in the initiation and progression of CRC (1–3). Large-scale sequencing analyses have identified numerous recurrently mutated genes and chromosomal translocations (4–6). In addition, a number of microRNAs (miRNAs) have been previously reported to be associated with CRC (7,8). However, how miRNA changes contribute to colorectal tumorigenesis has not yet been defined. Further insight into these changes may identify potential biomarkers or therapeutic targets.

miRNAs are small non-protein coding RNA molecules that regulate gene expression (9,10). miRNAs are important in crucial cellular processes, including development, differentiation, proliferation, apoptosis and metabolism (11,12). miRNAs have been proven to interact with potential oncogenes or tumor suppressors, and a number of miRNAs are differentially expressed in normal and neoplastic tissues and in tumors. The differential expression of miRNA has been previously evaluated as a predictive signature of cancer (13–17).

The present study investigated the expression profile of miRNAs in the serum of CRC patients. In total, 19 miRNAs were identified by high-throughput sequencing. A network analysis was performed based on a computational approach to identify associations between CRC and miRNAs. The network analysis of miRNA-target genes may aid in identifying altered miRNA regulatory networks that are involved in tumor pathogenesis.

## Materials and methods

### Samples and RNA extraction

Primary tumor and neighboring non-tumorous tissues were obtained from five CRC patients. All samples were collected according to procedures approved by the Institutional Review Board of the Guangzhou Medical University (Guangzhou, China) and individuals can not be identified from data or images included in the present study.

Tissue samples were flash frozen in liquid nitrogen and stored at −80°C until nucleic acid extraction. In total, 200 mg of fresh frozen tissues were used to isolate total RNA by phenol extraction (TRIzol reagent; Invitrogen, Life Technologies, Carlsbad, CA, USA). RNA concentration and purity were controlled by A NanoDrop spectrophotometer (Thermo Fisher Scientific, Waltham, MA, USA). The Agilent 2100 Bioanalyzer (Agilent Technologies, Inc., Santa Clara, CA, USA) was used to measure the quantity, integrity and purity of the small RNA. Patients provided written informed consent and the study was approved by the ethics committee of the Third Hospital of the Guangzhou Medical University.

### miRNA sequencing and sequence analysis

The miRNA expression profile was determined using the Ion Torrent PGM™ sequencer (Life Technologies). Briefly, the Ion Total RNA-Seq kit v2 (Life Technologies) was used to make small RNA libraries that preserve strand information. The templates were prepared using the Ion OneTouch™ system (Life Technologies), and small RNA analysis was performed using the Ion PGM™ sequencer. The miRNA sequences were analyzed by the Torrent Suite software (Life Technologies), and by miRWalk (http://mirwalk.uni-hd.de) and miRBase (http://www.mirbase.org). In addition, gene ontology (GO) and pathway analyses were performed using several tools that identify pathways and GO based on data sets from sequencing with the intent to identify miRNA-related genes and pathways. The tools include Ingenuity Systems Bioinformatics Software (Ingenuity Systems, Inc., Redwood City, CA, USA), FatiGO (http://fatigo.bioinfo.cnio.es) and Gene Expression Omnibus (http://www.ncbi.nlm.nih.gov/gds).

## Results

### Altered miRNA expression in CRC patients

The miRNA profiles of five pairs of solid tumor and adjacent tissues were compared. In total, 16 miRNAs exhibited higher expression in the solid tumor tissues, while three miRNAs exhibited higher expression profiles in the normal tissues; these are listed in Table I.

### GO and pathway analyses of miRNA target genes

**Figure 1:**
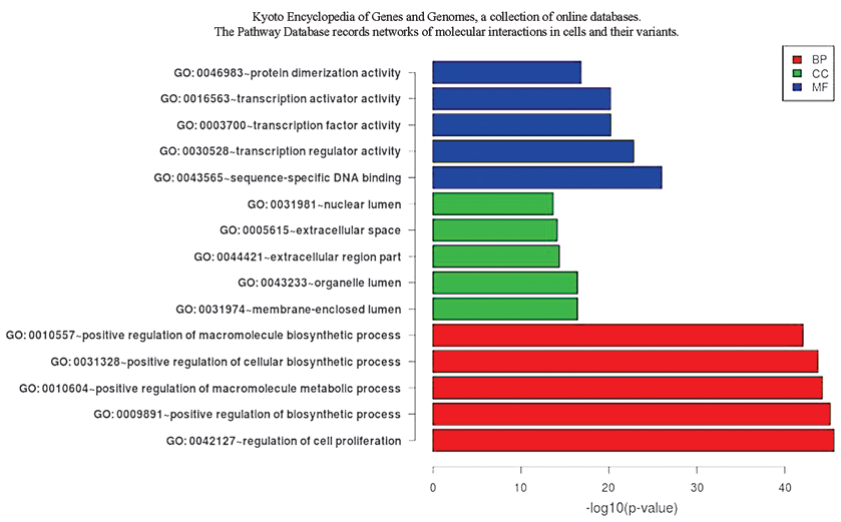
GO analysis. A number of target genes were found to be involved in cell proliferation and apoptosis. BP, biological process; CC, celluar component; MF, molecular function; GO, gene ontology.

**Figure 2:**
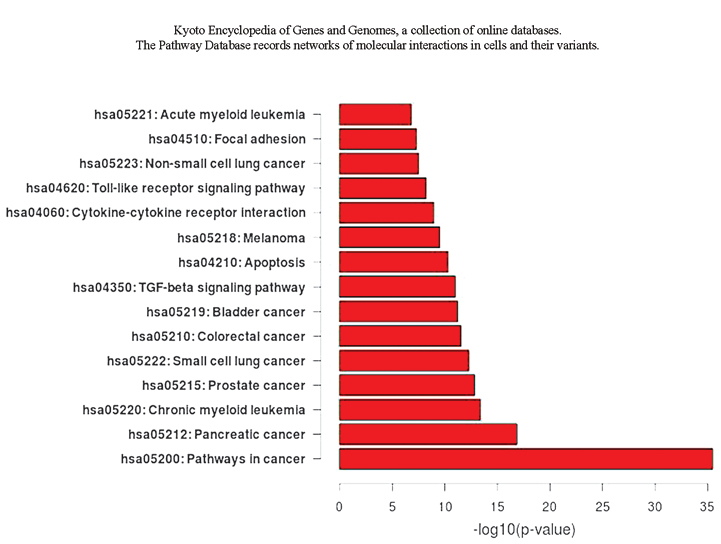
Pathway analysis. Transforming growth factor β and Toll-like signal pathways were found to be involved in cell proliferation and apoptosis.

The results of the GO analysis showed that a number of target genes are involved in cell proliferation and apoptosis. The pathway analysis showed that the transforming growth factor β and Toll-like signal pathways are also involved (Fig. 1 and 2).

## Discussion

miRNAs are small non-coding RNAs that enhance the cleavage or translational repression of specific mRNAs with recognition site(s) in the 3′-untranslated region. Since the identification of the miRNAs, several large-scale studies have compared the profiles of miRNA expression patterns between non-tumor and tumor tissues (18,19). A number of lines of evidence have shown that miRNA expression is predictive of outcome in patients with solid tumors. In lung cancer, low levels of let-7a have been associated with a short survival time following surgery. In addition, previous miRNA microarray expression profiling of tumors and paired non-tumorous tissues has been performed in colon cancer patients to identify miRNA expression patterns associated with outcome, and high levels of miR-21 have been found to be associated with a short overall survival time, independent of other factors (19,20).

In the current study, 19 miRNAs were identified by high-throughput sequencing. A network analysis was performed based on a computational approach to identify associations between CRC and miRNAs. The current study may be useful to identify cancer-specific signatures and potentially useful biomarkers for the diagnosis of CRC. The network analysis of miRNA-target genes may aid in identifying altered miRNA regulatory networks that are involved in tumor pathogenesis.

## Figures and Tables

**Table I:** Identification of miRNAs with higher expression profiles in normal or solid tumor tissues.

Solid tumor tissues	Normal adjacent tissues
hsa-miR-135b-5p	hsa-miR-100-5p
hsa-miR-146a-5p	hsa-miR-138-5p
hsa-miR-148b-3p	hsa-miR-191-5p
hsa-miR-17-5p	
hsa-miR-196a-5p	
hsa-miR-200a-3p	
hsa-miR-20a-5p	
hsa-miR-21-5p	
hsa-miR-223-3p	
hsa-miR-27a-5p	
hsa-miR-29b-3p	
hsa-miR-30e-5p	
hsa-miR-374b-5p	
hsa-miR-4787-5p	
hsa-miR-485-3p	
hsa-miR-660-5p	

miR/miRNA, microRNA; CRC, colorectal cancer.
